# Modeling the effects of consanguinity on autosomal and X-chromosomal runs of homozygosity and identity-by-descent sharing

**DOI:** 10.1093/g3journal/jkad264

**Published:** 2023-11-16

**Authors:** Daniel J Cotter, Alissa L Severson, Jonathan T L Kang, Hormazd N Godrej, Shai Carmi, Noah A Rosenberg

**Affiliations:** Department of Genetics, Stanford University, Stanford, CA, 94305, USA; Department of Genetics, Stanford University, Stanford, CA, 94305, USA; School of Math and Science, Singapore Polytechnic, 139651, Singapore; Department of Biology, Stanford University, Stanford, CA 94305, USA; Braun School of Public Health and Community Medicine, Hebrew University of Jerusalem, Jerusalem 9112102, Israel; Department of Biology, Stanford University, Stanford, CA 94305, USA

**Keywords:** coalescent theory, consanguinity, identity by descent, runs of homozygosity, X chromosome

## Abstract

Runs of homozygosity (ROH) and identity-by-descent (IBD) sharing can be studied in diploid coalescent models by noting that ROH and IBD-sharing at a genomic site are predicted to be inversely related to coalescence times—which in turn can be mathematically obtained in terms of parameters describing consanguinity rates. Comparing autosomal and X-chromosomal coalescent models, we consider ROH and IBD-sharing in relation to consanguinity that proceeds via multiple forms of first-cousin mating. We predict that across populations with different levels of consanguinity, (1) in a manner that is qualitatively parallel to the increase of autosomal IBD-sharing with autosomal ROH, X-chromosomal IBD-sharing increases with X-chromosomal ROH, owing to the dependence of both quantities on consanguinity levels; (2) even in the absence of consanguinity, X-chromosomal ROH and IBD-sharing levels exceed corresponding values for the autosomes, owing to the smaller population size and lower coalescence time for the X chromosome than for autosomes; (3) with matrilateral consanguinity, the relative increase in ROH and IBD-sharing on the X chromosome compared to the autosomes is greater than in the absence of consanguinity. Examining genome-wide SNPs in human populations for which consanguinity levels have been estimated, we find that autosomal and X-chromosomal ROH and IBD-sharing levels generally accord with the predictions. We find that each 1% increase in autosomal ROH is associated with an increase of 2.1% in X-chromosomal ROH, and each 1% increase in autosomal IBD-sharing is associated with an increase of 1.6% in X-chromosomal IBD-sharing. For each calculation, particularly for ROH, the estimate is reasonably close to the increase of 2% predicted by the population-size difference between autosomes and X chromosomes. The results support the utility of coalescent models for understanding patterns of genomic sharing and their dependence on sex-biased processes.

## Introduction

Autosomes and the X chromosome carry different signatures of population-genetic processes, owing both to differences in their mode of transmission and to demographic differences between males and females. Comparisons of autosomes and X chromosomes can therefore contribute to understanding genomic consequences of the different modes of transmission and of sex-biased and sex-specific processes, and many studies of autosomes and X chromosomes have considered empirical aspects of their population genetics in seeking such understanding (Wilkins and Marlowe 2006; Ramachandran *et al.* 2008; Bustamante and Ramachandran 2009; Ellegren 2009; Arbiza *et al.* 2014; Goldberg and Rosenberg 2015; Buffalo *et al.* 2016; Webster and Wilson Sayres 2016).

One set of population-genetic signatures that has the potential to be informative about sex-specific phenomena concerns features of genomic sharing: patterns in runs of homozygosity (ROH) and identity-by-descent (IBD) sharing on autosomes and the X chromosome (Buffalo *et al.* 2016; Cai *et al.* 2023). Recently, we have studied the distribution of the time to the most recent common ancestor ($T_{MRCA}$) for pairs of autosomal lineages and pairs of X-chromosomal lineages in diploid coalescent models under different types of consanguinity, considering coalescence of lineages within an individual and lineages in separate individuals (Severson *et al.* 2019, 2021; Cotter *et al.* 2021, 2022). This analysis finds that consanguinity decreases $T_{MRCA}$ both for lineage pairs in the same individual *and* for lineage pairs in individuals in different mating pairs. Further, because genomic sharing at a locus increases with decreasing $T_{MRCA}$, consanguinity increases genomic sharing both within (ROH) and between individuals (IBD) (Severson *et al.* 2019). Considering autosomal and X-chromosomal systems separately, relationships between consanguinity levels and $T_{MRCA}$ values produce predictions about relative values of autosomal and X-chromosomal ROH and IBD—with consanguinity that proceeds via matrilateral first-cousin mating reducing X-chromosomal coalescence times to a greater extent than patrilateral first-cousin mating (Cotter *et al.* 2021, 2022).

Here, we study the connections between autosomal and X-chromosomal $T_{MRCA}$ and features of X-chromosomal and autosomal ROH and IBD. Adding consideration of recombination to our diploid coalescent models, we examine predictions that compare X-chromosomal ROH to X-chromosomal IBD-sharing, X-chromosomal ROH to autosomal ROH, and X-chromosomal IBD-sharing to autosomal IBD-sharing. We consider human population-genetic data on ROH and IBD in a set of populations with consanguinity rates documented from demographic studies, using the results to understand effects of different forms of consanguinity on genomic sharing.

## Theory

### No consanguinity

#### Model

To derive expectations about features of genomic sharing on the autosomes and the X chromosome, we first consider a diploid, constant-sized population with *N* male–female mating pairs. We assume that recombination is constant across the autosomes and occurs at a per-Morgan rate proportional to the number of generations, 2g, separating two sampled alleles. To account for differences between the X-chromosome and the autosomes, we assume 4N autosomes for every 3N X chromosomes and a scaled X-chromosomal recombination rate $\frac{2}{3}$ that of the autosomes—because recombination occurs only in females and X-chromosomes are in females two thirds of the time (Hedrick 2007).

The calculations in this section derive from work on coalescent theory and its relationship to genomic sharing (Palamara *et al.* 2012; Carmi *et al.* 2014; Browning SR and Browning BL 2015). In general, this type of theoretical computation combines the coalescence-time distribution and a random variable that describes the length distribution of a segment given a specified time to the MRCA. Below, we derive the ratio of the expectation of total sharing on the X chromosome to the expectation of total sharing on the autosomes.

#### Expected X-chromosomal:autosomal total genomic sharing

In the absence of consanguinity, we derive a prediction for the ratio of the expected fraction of the X chromosome that lies in IBD segments and the corresponding expected fraction of the autosomal genome that lies in IBD segments. For a population with a demographic model whose parameterization is abbreviated by a quantity *θ* and whose recombination process has parameterization *ρ*, Palamara *et al.* (2012) specified the probability density function p(ℓ∣θ,ρ) that a specific locus is spanned by an IBD segment of a specific genetic length ℓ. For the closed interval R=[u,v], the probability that a locus is spanned by an IBD segment with length in *R* is


$$P_{R}(\ell |\theta ,\rho )=\int_{u}^{v}p(\ell |\theta ,\rho )d\ell .$$

Palamara *et al.* (2012) separated p(ℓ∣θ,ρ) into two terms by marginalizing over the number of generations to the most recent common ancestor, measured in discrete time as a random variable $g_{mrca}$. Following their equations (1) and (2),


(1) $$p(\ell |\theta ,\rho )=\sum_{g=1}^{\infty }p(g_{mrca}=g|\theta )\;p(\ell |g_{mrca}=g,\rho ).$$

The term $p(g_{mrca}=g|\theta )$ is the coalescence-time distribution, which for a constant-sized population (parameterizing *θ* with a population size of $N_{e}$ lineages) is a geometric random variable with rate $1/N_{e}$. The term $p(\ell |g_{mrca}=g,\rho )$ is the probability density of the length of a segment around a randomly chosen locus with coalescence time $g_{mrca}=g$.

Treating the distance from the locus to a recombination event as exponentially distributed, so that the total length of a shared segment between two lineages is the sum of two exponential random variables—the distance to the next recombination on the left plus the distance to the next recombination on the right—and measuring R=[u,v] in centimorgans, they obtained in their equation (4):


(2) $$P_{R}\left(\ell |\theta =N_{e},\rho =\frac{t}{50}\right)=\int_{0}^{\infty }\left[\frac{e^{-\frac{t}{N_{e}}}}{N_{e}}\int_{u}^{v}Erl_{2}\left(\ell ;\frac{t}{50}\right)d\ell \right]dt.$$

The first term is $p(t_{mrca}=t|\theta )$ (note the switch to continuous time, substituting the discrete, geometric $g_{mrca}$ by the continuous, exponential $t_{mrca}$ still measured in units of generations). The second, $p(\ell |t_{mrca}=t,\rho )$, is an Erlang density $(t/50{)}^{2}\ell e^{-\ell t/50}$ (Johnson *et al.* 1994, pg. 552) with shape parameter 2 and rate parameter $\rho =\frac{t}{50}$ centimorgans. With R=[u,∞), representing segments of size *u* centimorgans or greater, the inner integral gives (Palamara *et al.* 2012)


$$P_{R}\left(\ell |\theta =N_{e},\rho =\frac{t}{50}\right)=\int_{0}^{\infty }\left[\frac{e^{-\frac{t}{N_{e}}}}{N_{e}}\left(1+\frac{ut}{50}\right)e^{\frac{-ut}{50}}\right]dt.$$

For the autosomes, we set $N_{e}=4N$ for a population size of 4N autosomal lineages:


(3) $$\begin{array}{r} P_{R}^{A}\left(\ell |\theta =4N,\rho =\frac{t}{50}\right)=\int_{0}^{\infty }\left[\frac{e^{-\frac{t}{4N}}}{4N}\left(1+\frac{ut}{50}\right)e^{-\frac{ut}{50}}\right]dt=\frac{25(25+4Nu)}{(25+2Nu{)}^{2}} \end{array}.$$

Similarly, for the X chromosome, we set $N_{e}=3N$ for the reduced number of X-chromosomal lineages. We rescale the $\rho =\frac{t}{50}$ centimorgans from equation (2) by $\frac{2}{3}$, giving $\rho =\frac{t}{75}$, to account for the reduced recombination rate:


(4) $$\begin{array}{r} P_{R}^{X}\left(\ell |\theta =3N,\rho =\frac{t}{75}\right)=\int_{0}^{\infty }\left[\frac{e^{-\frac{t}{3N}}}{3N}\left(1+\frac{ut}{75}\right)e^{-\frac{ut}{75}}\right]dt=\frac{25(25+2Nu)}{(25+Nu{)}^{2}} \end{array}.$$

The expected fraction *f* of the genome that lies in IBD segments in length interval *R* is $E_{R}[f|\theta ,\rho ]=P_{R}(\ell |\theta ,\rho )$ (Palamara *et al.* 2012, equation 9). Using equations (3) and (4), we can express the ratio of the expected fraction of the X chromosome that lies in IBD segments with length in R=[u,∞) and the expected fraction of the autosomes that lies in IBD segments with length in R=[u,∞):


$$\begin{array}{r} \frac{E_{R}^{X}[f|\theta =3N,\rho =\frac{t}{75}]}{E_{R}^{A}[f|\theta =4N,\rho =\frac{t}{50}]}=\frac{P_{R}^{X}(\ell |\theta =3N,\rho =\frac{t}{75})}{P_{R}^{A}(\ell |\theta =4N,\rho =\frac{t}{50})}=\frac{(25+2Nu{)}^{3}}{\left(25+Nu{)}^{2}(25+4Nu\right)} \end{array}.$$

Taking N→∞, we obtain


(5) $$\lim_{N\to \infty }\frac{E_{R}^{X}[f|\theta =3N,\rho =\frac{t}{75}]}{E_{R}^{A}[f|\theta =4N,\rho =\frac{t}{50}]}=2.$$

Because this limit does not depend on the lower limit of interval *R*, the population-size difference for X chromosomes and autosomes gives rise to a prediction that, irrespective of the interval *R*, for large *N*, the fraction of the X chromosome that lies in IBD segments with lengths in *R* is twice the corresponding fraction for autosomes.

A similar argument holds for ROH. A pair of lineages in a single individual is inherited from two lineages in two separate individuals in the previous generation. In an infinite population without consanguinity, the two lineages in the parental generation represent two independent draws from the population. Hence, the genomic sharing of the parental lineages follows the behavior we have described for IBD-sharing. To produce two lineages in the offspring, one additional generation of recombination occurs; however, the probability that a recombination event changes the IBD status of two lineages in one generation is small, so that ROH behavior in the offspring closely follows the IBD behavior of the parents. We can conclude that, as we found for IBD segments, the fraction of the X chromosome that lies in ROH segments with lengths in *R* is equal to twice the corresponding fraction for autosomes.

### Consanguinity

#### Model

**Fig. 1.**
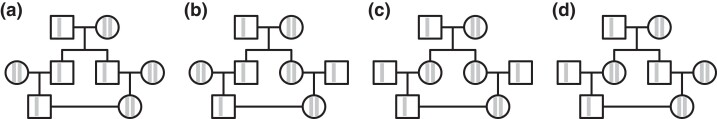
X chromosomes in first-cousin mating schemes. A) Patrilateral-parallel. B) Patrilateral-cross. C) Matrilateral-parallel. D) Matrilateral-cross.

We have previously studied the effects of first-cousin consanguinity on coalescence times (Cotter *et al.* 2021, 2022). Under a coalescent model, extending work of Campbell (2015) and Severson*et al.* (2019, 2021), we considered a population of *N* diploid mating pairs, labeling individuals by sex. In each generation, a fraction $c_{1}$ of the mating pairs are consanguineous, with a specific mixture of different types of first-cousin consanguinity ($c_{pp}$ for patrilateral-parallel, $c_{pc}$ for patrilateral-cross, $c_{mp}$ for matrilateral-parallel, $c_{mc}$ for matrilateral-cross—see Fig. 1). Under the model, we computed limiting distributions for pairwise values of the time to the MRCA ($T_{MRCA}$) for two autosomal lineages in the same individual, two X-chromosomal lineages in the same individual, two autosomal lineages in different individuals, and two X-chromosomal lineages in different individuals (Table 1). The results rely on N→∞ limits via the separation-of-time-scales method of Möhle (1998), in which a “fast” process induces a nonzero probability of instantaneous coalescence; the remaining coalescence occurs by a “slow” process that takes a positive amount of time. They can be regarded as approximate for finite populations.

**Table 1.** Limiting cumulative distribution functions for coalescence times for two X-chromosomal and two autosomal lineages sampled within- and between-individuals.

	Chromosome	Cumulative distribution	Equation from Cotter *et al.* (2022)
Within-individuals (ROH)	Autosomes	$1-\frac{1-\frac{c_{1}}{4}}{1-\frac{3}{16}c_{1}}e^{-\frac{t}{4N}\left(\frac{1}{1-\frac{3}{16}c_{1}}\right)}$	C2
	X	$1-\frac{1-\frac{c_{mp}}{2}-\frac{c_{mc}}{2}}{1-\frac{5}{16}c_{mp}-\frac{3}{8}c_{mc}}e^{-\frac{t}{3N}\left(\frac{1+\frac{c_{mp}}{16}-\frac{c_{mc}}{8}}{1-\frac{5}{16}c_{mp}-\frac{3}{8}c_{mc}}\right)}$	37
Between-individuals (IBD)	Autosomes	$1-e^{-\frac{t}{4N}\left(\frac{1}{1-\frac{3}{16}c_{1}}\right)}$	C3
	X	$1-e^{-\frac{t}{3N}\left(\frac{1+\frac{c_{mp}}{16}-\frac{c_{mc}}{8}}{1-\frac{5}{16}c_{mp}-\frac{3}{8}c_{mc}}\right)}$	38

Equations are taken from Cotter *et al.* (2022).

ROH lengths are inversely related to within-individual coalescence times, and IBD lengths are inversely related to between-individual coalescence times. Hence, the $T_{MRCA}$ calculations in our model give rise to predictions about features of autosomal and X-chromosomal ROH and IBD. In general, because a population has fewer copies of an X-chromosomal locus than an autosomal locus, X-chromosomal coalescence times are smaller than autosomal coalescence times. We showed that in relation to values seen in a nonconsanguineous population, X-chromosomal within-individual coalescence times are reduced by consanguinity to a greater extent than are X-chromosomal between-individual coalescence times (Cotter *et al.* 2021, Table 1). Here, extending the results on genomic sharing from Palamara *et al.* (2012), we use the limiting coalescence-time distributions from Cotter *et al.* (2022) to derive theoretical predictions for features of ROH and IBD-sharing on the X-chromosome and the autosomes.

#### Expected X-chromosomal:autosomal total genomic sharing

To derive an expectation under our models of ROH and IBD-sharing with consanguinity, we begin by modifying equation (1), once again switching to continuous time, *t*. Because only the coalescence-time distribution depends on the underlying demography—the population size and the rates of first-cousin consanguinity—it suffices to apply $p(t_{mrca}=t|\theta )$ and *ρ* in different versions of the demographic model.

It is convenient to begin with between-individual coalescence times and IBD-sharing. Using the coalescence-time distributions in Table 1, the time to the most recent common ancestor for two lineages in two separate individuals follows a coalescent with the population size scaled based on the rates for the different types of consanguinity. Converting the cumulative distributions in Table 1 to their probability density functions and annotating $\theta =\{4N,c_{1}\}$ and $\theta =\{3N,c_{mp},c_{mc}\}$ for the autosomes and X chromosome, respectively, we have


(6) $$p_{A}(t_{mrca}=t|\theta =\{4N,c_{1}\})=\frac{1}{4N\left(1-\frac{3}{16}c_{1}\right)}e^{-\frac{t}{4N}\left(\frac{1}{1-\frac{3}{16}c_{1}}\right)},$$

(7) $$p_{X}(t_{mrca}=t|\theta =\{3N,c_{mp},c_{mc}\})=\frac{1+\frac{c_{mp}}{16}-\frac{c_{mc}}{8}}{3N\left(1-\frac{5}{16}c_{mp}-\frac{3}{8}c_{mc}\right)}e^{-\frac{t}{3N}\left(\frac{1+\frac{c_{mp}}{16}-\frac{c_{mc}}{8}}{1-\frac{5}{16}c_{mp}-\frac{3}{8}c_{mc}}\right)}.$$

We solve for the expected fraction of the autosomes and the X chromosome appearing in IBD segments (using Palamara *et al.* 2012, equation 9). For the autosomes, using equation (6) for the coalescence-time distribution and parameterizing recombination by $\rho =\frac{t}{50}$, the expected fraction of the autosomes shared identically by descent in a population with *N* mating pairs and proportion $c_{1}=c_{pp}+c_{pc}+c_{mp}+c_{mc}$ of first-cousin mating per generation is


(8) $$\begin{array}{lll} E_{R,b}^{A}\left[f|\theta =\{4N,c_{1}\},\rho =\frac{t}{50}\right] & = & \int_{0}^{\infty }p_{A}(t|\theta )\times \left[\left(1+\frac{ut}{50}\right)e^{-\frac{ut}{50}}\right]dt\\ & = & \frac{25\left[25+4N\left(1-\frac{3}{16}c_{1}\right)u\right]}{{\left[25+2N\left(1-\frac{3}{16}c_{1}\right)u\right]}^{2}}. \end{array}$$

Here, we have written $E_{R,b}^{A}[f]$ for the expected fraction of the autosomal genome shared in R∈[u,∞) between individuals (with the subscript *b* differentiating this quantity from a corresponding expectation *within* individuals). For the X chromosome, using equation (7) for coalescence times and $\rho =\frac{t}{75}$ for recombination, we have


(9) $$\begin{array}{rl} E_{R,b}^{X}\left[f|\theta =\{3N,c_{mp},c_{mc}\},\rho =\frac{t}{75}\right] & =\int_{0}^{\infty }p_{X}(t|\theta )\times \left[\left(1+\frac{ut}{75}\right)e^{-\frac{ut}{75}}\right]dt\\ & =\frac{25\left[25+2N\left(\frac{1-\frac{5}{16}c_{mp}-\frac{3}{8}c_{mc}}{1+\frac{c_{mp}}{16}-\frac{c_{mc}}{8}}\right)u\right]}{{\left[25+N\left(\frac{1-\frac{5}{16}c_{mp}-\frac{3}{8}c_{mc}}{1+\frac{c_{mp}}{16}-\frac{c_{mc}}{8}}\right)u\right]}^{2}}. \end{array}$$

Next, relying on the within-individual coalescence-time distributions for two lineages, we use a similar framework to evaluate the expected fraction of the genome that lies in runs of homozygosity. A point mass exists for the probability of instantaneous coalescence at t=0 in the cumulative distributions in Table 1: $\left(\frac{c_{1}}{16})/(1-\frac{3}{16}c_{1}\right)$ for the autosomes and $\left(\frac{3}{16}c_{mp}+\frac{1}{8}c_{mc})/(1-\frac{5}{16}c_{mp}-\frac{3}{8}c_{mc}\right)$ for the X chromosome, obtained by substituting t=0 in the cumulative distributions. We express the expected fractions of the autosomes and X chromosome that lie in ROH using the instantaneous coalescence probabilities; for noninstantaneous coalescence, we follow equations (6) and (7).

We write $E_{R,w}^{A}[f]$ for the expected fraction of the genome shared within individuals in the length interval R∈[u,∞). For the autosomes, with recombination parameterized by $\rho =\frac{t}{50}$, we have


(10) $$\begin{array}{rl} E_{R,w}^{A}\left[f|\theta =\{4N,c_{1}\},\rho =\frac{t}{50}\right] & =\frac{\frac{c_{1}}{16}}{1-\frac{3}{16}c_{1}}+\left(1-\frac{\frac{c_{1}}{16}}{1-\frac{3}{16}c_{1}}\right)\\ & \;\times \int_{0}^{\infty }p_{A}(t|\theta )\times \left[\left(1+\frac{ut}{50}\right)e^{-\frac{ut}{50}}\right]dt\\ & =\frac{\frac{c_{1}}{16}}{1-\frac{3}{16}c_{1}}+\frac{1-\frac{c_{1}}{4}}{1-\frac{3}{16}c_{1}}\\ & \;\times \left(\frac{25\left[25+4N\left(1-\frac{3}{16}c_{1}\right)u\right]}{{\left[25+2N\left(1-\frac{3}{16}c_{1}\right)u\right]}^{2}}\right). \end{array}$$

Similarly, for the X chromosome, with $\rho =\frac{t}{75}$, we have


(11) $$\begin{array}{rl} E_{R,w}^{X}\left[f|\theta =\{3N,c_{mp},c_{mc}\},\rho =\frac{t}{75}\right] & =\frac{\frac{3}{16}c_{mp}+\frac{c_{mc}}{8}}{1-\frac{5}{16}c_{mp}-\frac{3}{8}c_{mc}}\\ & \;+\left(1-\frac{\frac{3}{16}c_{mp}+\frac{c_{mc}}{8}}{1-\frac{5}{16}c_{mp}-\frac{3}{8}c_{mc}}\right)\\ & \;\times \int_{0}^{\infty }p_{X}(t|\theta )\times \left[\left(1+\frac{ut}{75}\right)e^{-\frac{ut}{75}}\right]dt\\ & =\frac{\frac{3}{16}c_{mp}+\frac{c_{mc}}{8}}{1-\frac{5}{16}c_{mp}-\frac{3}{8}c_{mc}}\\ & \;+\frac{1-\frac{c_{mp}}{2}-\frac{c_{mc}}{2}}{1-\frac{5}{16}c_{mp}-\frac{3}{8}c_{mc}}\\ & \;\times \left(\frac{25\left[25+2N\left(\frac{1-\frac{5}{16}c_{mp}-\frac{3}{8}c_{mc}}{1+\frac{c_{mp}}{16}-\frac{c_{mc}}{8}}\right)u\right]}{{\left[25+N\left(\frac{1-\frac{5}{16}c_{mp}-\frac{3}{8}c_{mc}}{1+\frac{c_{mp}}{16}-\frac{c_{mc}}{8}}\right)u\right]}^{2}}\right). \end{array}$$

**Fig. 2.**
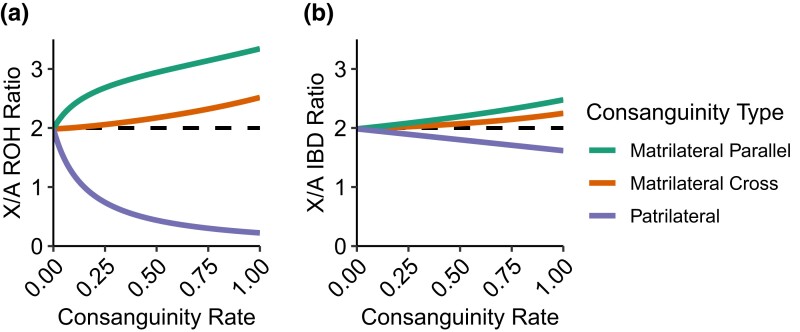
Expected ROH and IBD-sharing on the X chromosome relative to the autosomes as a function of consanguinity. A) ROH. B) IBD. For ROH, the ratio is calculated as equation (11)/equation (10), and for IBD, it is calculated as equation (9)/equation (8). In both cases, N=500, u=5 cM, and only one type of consanguinity is varied at a time while holding the others at 0. Patrilateral-parallel and patrilateral-cross consanguinity have the same effect.

In Fig. 2, we explore the effects of the various types of first-cousin consanguinity on the ratio between X-chromosomal and autosomal ROH and IBD. To clarify the effects of the types of consanguinity one at a time, we plot the ratio of equation (11) to equation (10) for ROH (Fig. 2A) and equation (9) to equation (8) for IBD (Fig. 2B). For illustration, we choose values N=500 for the population size and u=5 cM for the minimal segment length, varying only one consanguinity rate at a time. A population in which multiple consanguinity values are positive combines the various individual scenarios.

Both for IBD and for ROH, increasing the first-cousin consanguinity shifts the X:autosomal ratio away from the expectation of 2 given in equation (5). Patrilateral consanguinity decreases this ratio below 2, whereas matrilateral consanguinity increases it above 2, with matrilateral-parallel producing a greater increase than matrilateral-cross. The effect of consanguinity on the ROH ratios (Fig. 2A) has magnitude greater than the effect on corresponding IBD ratios (Fig. 2B).

These patterns accord with the large-*N* limits for the ROH and IBD X:autosomal ratios. For ROH, the N→∞ limit of the ratio of equation (11) to equation (10) is


(12) $$\lim_{N\to \infty }\frac{E_{R,w}^{X}\left[f|\theta =\{3N,c_{mp},c_{mc}\},\rho =\frac{t}{75}\right]}{E_{R,w}^{A}\left[f|\theta =\{4N,c_{1}\},\rho =\frac{t}{50}\right]}=\frac{\left(1-\frac{3}{16}c_{1}\right)\left(\frac{3}{16}c_{mp}+\frac{c_{mc}}{8}\right)}{\frac{c_{1}}{16}\left(1-\frac{5}{16}c_{mp}-\frac{3}{8}c_{mc}\right)},$$

recalling that $c_{1}$ is the sum of the rates of all four types of first-cousin consanguinity, $c_{pp}+c_{pc}+c_{mp}+c_{mc}$. Varying $c_{pp}+c_{pc}$ in (0,1] and holding $c_{mp}=c_{mc}=0$, the limiting ratio is 0: patrilateral consanguinity produces no ROH on the X chromosome but a positive level of ROH on the autosomes. For $c_{mp}\in (0,1]$ and all other consanguinity rates set to 0, the limiting ratio varies from minimum 3 ($c_{mp}\to 0$) to maximum $\frac{39}{11}\approx 3.545$ ($c_{mp}=1$). For $c_{mc}\in (0,1]$ and all other consanguinity rates set to 0, the limiting ratio is 2 at the minimum ($c_{mc}\to 0$) and $\frac{13}{5}=2.6$ at the maximum ($c_{mc}=1$). Note that the limiting function is undefined for $c_{1}=0$.

Similarly for IBD, the N→∞ limit of the ratio of equation (9) to equation (8) is


(13) $$\lim_{N\to \infty }\frac{E_{R,b}^{X}\left[f|\theta =\{3N,c_{mp},c_{mc}\},\rho =\frac{t}{75}\right]}{E_{R,b}^{A}\left[f|\theta =\{4N,c_{1}\},\rho =\frac{t}{50}\right]}=2\left[\frac{1-\frac{3}{16}c_{1}}{\left(\frac{1-\frac{5}{16}c_{mp}-\frac{3}{8}c_{mc}}{1+\frac{c_{mp}}{16}-\frac{c_{mc}}{8}}\right)}\right].$$

At $c_{1}=0$, this limit is 2, as in the case without consanguinity. If $c_{pp}+c_{pc}=1$ and the other rates are held at 0, then the limiting ratio is $\frac{13}{8}=1.625$. If $c_{mp}=1$, then the limit is $\frac{221}{88}\approx 2.511$. If $c_{mc}=1$, then it is $\frac{91}{40}=2.275$.

## Data analysis

### Data

#### Demographic data

We consider a large demographic study that counted consanguineous pairs of various types—including first-cousin consanguineous pairs—among parents of newborns born in Israel 1955–1957 (Goldschmidt *et al.* 1960). For each of a series of Jewish populations, among first-cousin mating pairs, Goldschmidt *et al.* (1960) tabulated numbers of patrilateral-parallel, patrilateral-cross, matrilateral-parallel, and matrilateral-cross cousin pairs. As a fraction of all mating pairs, we denote these quantities $c_{pp}$, $c_{pc}$, $c_{mp}$, and $c_{mc}$, respectively.

For nine populations that overlap between the demographic data of Goldschmidt *et al.* (1960) and genetic data used by Kang *et al.* (2016) and Severson *et al.* (2019), the rates $c_{pp}$, $c_{pc}$, $c_{mp}$, and $c_{mc}$ appear in Table 2. In all nine populations, matrilateral consanguinity $c_{mp}+c_{mc}$ is nonzero, so that consanguinity influences X-chromosomal coalescence times, and hence ROH and IBD-sharing for both autosomes and X chromosomes.

**Table 2.** Rates of the four different first-cousin mating types across 9 Jewish populations. As in Kang *et al.* (2016), the population listed as “Sephardi” corresponds to the “Turkey” population in Goldschmidt *et al.* (1960); the population listed as “Iranian” corresponds to the “Persia” population.

	Frequency of first-cousin mating pairs (%)			
Population	Patrilateral parallel ($c_{pp}$)	Patrilateral cross ($c_{pc}$)	Matrilateral parallel ($c_{mp}$)	Matrilateral cross ($c_{mc}$)
Ashkenazi	0.507	0.296	0.465	0.084
Iranian	4.215	2.576	4.684	4.450
Iraqi	4.483	2.759	5.724	3.448
Libyan	2.013	2.685	0.671	0.671
Moroccan	0.794	0.794	1.984	1.587
Sephardi	0.329	0.494	0.988	1.318
Syrian	0.985	0.493	0.985	1.232
Tunisian	2.685	1.342	4.027	2.685
Yemenite	3.347	1.071	1.874	1.606

Values are calculated from Tables 1 and 3 of Goldschmidt *et al.* (1960) as fractions of all mating pairs that are first-cousin pairs of particular types (omitting one double-first-cousin pair from both of its constituent categories of first-cousin pairs).

#### Autosomal genetic data

For the autosomes, we used genetic data from Kang *et al.* (2016), consisting of 202 Jewish individuals from 18 populations and 2,903 non-Jewish individuals from 123 populations, with 257,091 SNPs. These data are a merged data set constructed from data from Behar *et al.* (2013) and from the HGDP-CEPH and HapMap panels, as studied by Verdu *et al.* (2014). From these data, as in Severson *et al.* (2019), we consider the subset of 202 individuals from 18 Jewish populations, using the non-Jewish individuals only for phasing. These are the same individuals and same genotypes used by Kang *et al.* (2016) to call autosomal ROH segments and by Severson *et al.* (2019) to call autosomal IBD segments. We use the autosomal ROH segments directly from Kang *et al.* (2016), but we perform our own calls of autosomal IBD segments with updates of the method used by Severson *et al.* (2019).

#### X-chromosomal genetic data

For the X chromosome, we used genotypes from Behar *et al.* (2013). Beginning with 1,774 individuals and 32,823 SNPs, we first removed SNPs that were completely missing or monoallelic. Next, in individuals labeled as males, we verified the label by assessing heterozygosity of X-chromosomal genotypes, converting the small number of heterozygous genotypes to missing data (Supplementary Fig. S1). We then removed, in sequence, SNPs missing in a large number of individuals (>200) and individuals missing a large number of SNPs (>2,500).

After processing, the data contained 1,647 individuals (1,227 males, 420 females) and 13,052 SNPs, comparable to the SNP density in the autosomal data (Supplementary Fig. S1). This collection contains 168 Jewish individuals from 18 populations (Supplementary Table S1) and 1,479 non-Jewish individuals. We focus on the Jewish individuals for our analysis and include non-Jewish individuals only for phasing of both autosomal and X-chromosomal genotypes.

### Methods

#### ROH

ROH lengths for the autosomes were taken directly from Kang *et al.* (2016). These ROH lengths were classified by Kang *et al.* (2016) into 3 length classes; for our analyses, we used the total length of all classes.

To measure ROH lengths for the X chromosome, we followed the procedure of Kang *et al.* (2016), with four modifications to account for differences between the X chromosome and autosomes. (1) In calculating sample allele frequencies for the X chromosome for each SNP in each population, we calculated the allele frequency with males contributing one allele and females contributing two. As in Kang *et al.* (2016), we performed 40 Bernoulli draws with this “true” allele frequency to obtain a sample allele frequency. This procedure reduces sample-size effects on ROH calls. (2) We used only females for identifying ROH, as males have only a single X chromosome. (3) For overlapping windows of 30 SNPs, Kang *et al.* (2016) calculated a log-likelihood (LOD) score to determine if windows were autozygous. The distribution of all LOD scores in a population was then used to set the threshold for calling ROH in the population. For consistency, and because identification of LOD score cutoffs for X-chromosomal data is more uncertain than for the autosomes due to a smaller number of X-chromosomal ROH available in our relatively small sample size, we used the autosomal LOD score cutoffs from Kang *et al.* (2016) rather than using X-chromosome-specific LOD scores (Supplementary Table S2). (4) Due to the smaller amount of data available for subdividing ROH into length classes, we did not attempt to determine length classes for X-chromosomal ROH.

For each population, we summarized ROH lengths on the autosomes and X chromosome as the mean total proportion of the genome contained in ROH. First, we calculated the mean total ROH length as the sum of the lengths of ROH segments across all individuals in a population divided by the total number of individuals, considering only females for the X chromosome. For autosomes, we normalized this quantity by 2,881.03 Mb for the combined length of chromosomes 1 through 22; for the X chromosome, we used 155.27 Mb. We base these lengths on human genome assembly GRCh37, as reported in the UCSC Genome Browser (Kent *et al.* 2002).

#### IBD-sharing

We calculated autosomal IBD-sharing using the data from Kang *et al.* (2016). For each chromosome, we phased the full data set of 3,105 individuals using Beagle 5.1 (Browning SR and Browning BL 2007) and default parameters (burnin=6, iterations=12, phase-states=280, impute=false, ne=1,000,000, window=40.0, overlap=4.0, seed=-99,999), with the GRCh37 genetic map for the map parameter (as provided with Beagle). We then considered the subset of 202 individuals in 18 Jewish populations, calling IBD segments using Refined IBD (Browning BL and Browning SR 2013) with default parameters (window=40.0, lod=3.0, length=1.5, trim=0.15) and the map used for phasing. Our autosomal IBD calculations employed the method and data of Severson *et al.* (2019), except that we used a newer Beagle version and called IBD-sharing only on the subset of Jewish individuals rather than the whole sample.

For the X chromosome, we used data from the full 1,647 individuals (including the 168 Jewish individuals). We recoded alleles in males as pseudodiploid, as needed by Beagle 5.1 and Refined IBD. We then phased the 1,647 individuals with Beagle 5.1 using the same parameters and map as used for the autosomes. In the phased data, considering only the Jewish populations, we calculated IBD segments using Refined IBD in the same manner as for the autosomes. We then removed all duplicate IBD segments that resulted from pseudodiploid coding in males.

In each population, we summarized IBD-sharing as the mean total IBD proportion. That is, for each pair of individuals, we called IBD-sharing on the autosomes between four pairs of haplotypes, two in each individual in the pair. On the X chromosome, IBD comparisons considered one pair of haplotypes for pairs of males, two pairs for a male and a female, and four pairs for pairs of females. Thus, we divided the total IBD length between two individuals—summing across pairs of X chromosomes, one from one individual and one from the other—by one (two haplotypes), two (three haplotypes), or four (four haplotypes). We calculated mean total IBD length as the mean across pairs of individuals after accounting for the number of pairwise haplotype comparisons. We then normalized this quantity, using the same genomic lengths as for ROH, to determine population-wise mean IBD proportions.

#### Population subsets

Because individuals with available X-chromosomal data represent a subset of the individuals with available autosomal data, in the following analyses, we used only a subset of the 18 populations. In particular, when comparing autosomal and X-chromosomal ROH, we considered only 13 populations, omitting 5 populations (Cochin, Georgian, Libyan, Mumbai, Syrian) for which no females and hence no X-chromosomal ROH calls were available (Supplementary Table S1).

### Results

Our theoretical results predict an increased proportion of ROH and IBD on the X chromosome relative to the autosomes as well as a positive relationship between IBD-sharing and ROH: increasing consanguinity decreases $T_{MRCA}$ for two alleles within individuals as well as two alleles between individuals, in turn increasing both ROH and IBD-sharing (Severson *et al.* 2019; Cotter *et al.* 2021).

**Fig. 3.**
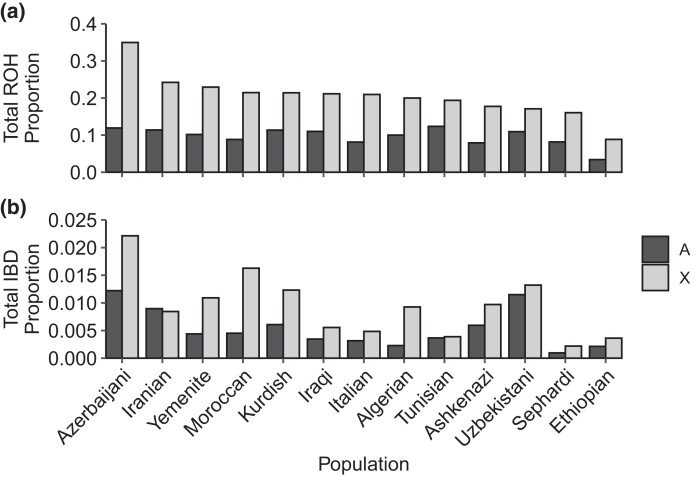
Proportion of autosomal and X-chromosomal ROH and IBD in each population. A) ROH. B) IBD. Populations are arranged in decreasing order by the proportion of the X-chromosomal genome lying in ROH.

Empirical ROH levels and IBD levels are greater on the X chromosome than on the autosomes (Fig. 3). The smaller total population size of the X chromosome, 3N compared to 4N in a population with equal sex ratio, produces lower coalescence times for the X chromosome, in turn giving rise to longer ROH and IBD segments.

We consider regressions of IBD proportions on ROH proportions, evaluating the coefficient of determination $R^{2}$ and the *P*-value for the null hypothesis of a regression slope of 0. In Supplementary Fig. S2, we plot the relationship between mean total IBD and ROH proportions in 13 populations, for both the autosomes and the X chromosome. Severson *et al.* (2019) previously performed this analysis for autosomes; here we compare autosomes and the X chromosome. In accord with the theoretical prediction, we see that IBD-sharing increases with ROH for the autosomes (Supplementary Fig. S2A; $R^{2}=0.27$), though not at the P=0.05 significance level (P=0.07). It also increases for the X chromosome (Supplementary Fig. S2B; $R^{2}=0.49$, P=0.008), for which the relationship is stronger.

**Fig. 4.**
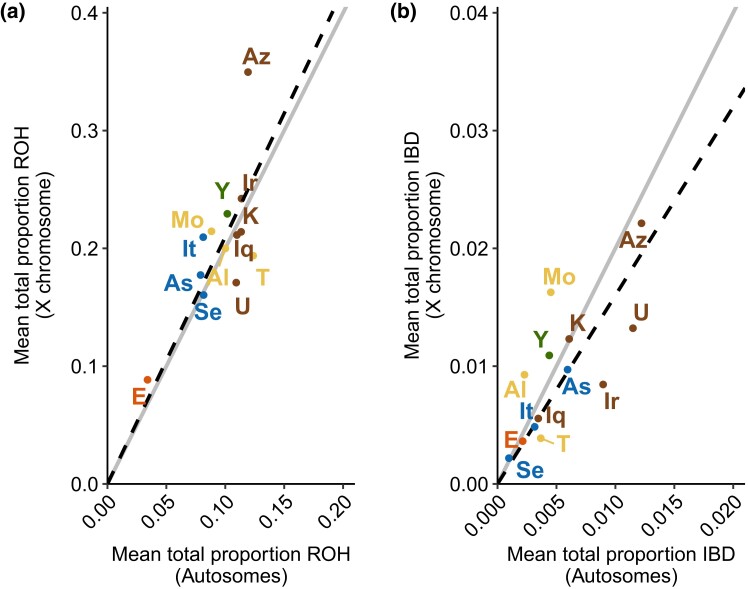
Mean genomic proportion contained in ROH and on the autosomes relative to the X chromosome. A) ROH. B) IBD. The solid line is the theoretical prediction y=2x. The dashed line represents a regression with intercept fixed at 0: y=2.10x ($R^{2}=0.96$, $P=6.13\times 10^{-10}$) A), y=1.60x ($R^{2}=0.87$, $P=1.45\times 10^{-6}$) B). Thirteen populations are color-coded by regional group as in Kang *et al.* (2016) and Severson *et al.* (2019): Ethiopian, orange; European, blue; Middle Eastern, brown; North African, yellow; Yemenite, green. Population labels: Al, Algerian; As, Ashkenazi; Az, Azerbaijani; E, Ethiopian; Iq, Iraqi; Ir, Iranian; It, Italian; K, Kurdish; Mo, Moroccan; Se, Sephardi; T, Tunisian; U, Uzbekistani; Y, Yemenite.

To explore the relationship between ROH patterns on the X chromosome and on autosomes, we next regress—with a fixed intercept of y=0—the mean ROH genomic fraction on the autosomes onto the corresponding mean for the X chromosome. X-chromosomal and total autosomal ROH are positively related (Fig. 4A; $R^{2}=0.96$, $P=6.13\times 10^{-10}$). The regression slope exceeds 2: for each 1% increase in total ROH on the autosomes, we see a 2.1% increase on the X chromosome. This greater increase for the X chromosome accords with the smaller X-chromosomal population size and reduced recombination rate—which inflate ROH for the X chromosome.

Next, having detected a relationship between total lengths in X-chromosomal and autosomal ROH, we compare genomic fractions of IBD-sharing. Fixing the regression intercept at y=0, X-chromosomal IBD increases with autosomal IBD (Fig. 4B; $R^{2}=0.87$, $P=1.45\times 10^{-6}$). A 1.6% increase in X-chromosomal IBD-sharing occurs for each 1% increase in autosomal IBD-sharing, consistent with the reduced population size of the X chromosome and its resulting reduction in coalescence times and increase in IBD segment length.

For the seven populations for which demographic estimates of consanguinity and genomic data are both available, we can compare the empirical ratio of the fractions of the X chromosome and autosomal genome that lie in ROH to a theoretical prediction. Inserting the consanguinity rates from Table 2 and a range of values of the number of mating pairs *N* from 500 to 50,000 into equations (11) and (10), we obtain predictions for the ratio of equations (11) and (10). The nontrivial patrilateral consanguinity in these populations, sometimes exceeding the matrilateral consanguinity, leads to predictions that lie below the ratio of 2 predicted from equation (5) in the case of no consanguinity (Table 3). The empirical ratios tend to be near but somewhat greater than the theoretical range, suggesting that while the differing numbers of autosomal genomes and X chromosomes and the effects of consanguinity in part explain differences in autosomal and X-chromosomal ROH, other factors also contribute.

**Table 3.** Theoretical and empirical ratios of the proportion of the X-chromosome to the proportion of the autosomal genome lying in ROH and IBD segments.

	ROH			IBD		
	Theoretical X:A ratio			Theoretical X:A ratio		
Population	Minimum	Maximum	Empirical X:A ratio	Minimum	Maximum	Empirical X:A ratio
Ashkenazi	1.541	1.935	2.247	1.542	1.991	1.633
Iranian	1.516	1.818	2.126	1.525	1.968	0.942
Iraqi	1.518	1.824	1.921	1.527	1.974	1.608
Moroccan	1.537	1.934	2.434	1.539	1.988	3.602
Sephardi	1.539	1.950	1.969	1.540	1.988	2.268
Tunisian	1.529	1.877	1.568	1.534	1.983	1.057

The theoretical ratio is the ratio of equations (11) and (10) for ROH and the ratio of equations (9) and (8) for IBD, inserting consanguinity rates from Table 2 and setting u=0.1 cM for the minimum size of ROH and IBD. We report the minimum and the maximum theoretical ratios achieved when varying *N* in the range [500,50,000]. The empirical ratio is calculated using the ROH and IBD proportions obtained via the Methods subsections on ROH and IBD, respectively.

For IBD, a similar calculation of the theoretical ratio of X-chromosomal and autosomal ROH, using equations (9) and (8), places the seven populations into similar ranges. This similarity illustrates the lesser effect of differences in consanguinity rates on the predicted ratio of X-chromosomal and autosomal IBD compared to the corresponding ratio for ROH (Fig. 2). Empirical IBD ratios tend to be farther from the predicted range than are empirical ROH ratios, indicating that the factors we have considered—population-size differences between the X chromosome and autosomes, and consanguinity rates—may be less determinative of IBD patterns than of ROH patterns.

## Discussion

This study has investigated the effect of consanguinity on X-chromosomal ROH and IBD-sharing. Under a coalescent model with consanguinity, we had previously obtained autosomal (Severson *et al.* 2019, 2021) and X-chromosomal (Cotter *et al.* 2021, 2022) distributions of coalescence times. Here, we have combined results on coalescence times with calculations based on properties of recombination to predict features of ROH and IBD-sharing under the model. We have also compared the predictions with empirical patterns in ROH and IBD-sharing in populations for which demographic measures of consanguinity have been reported.

For the coalescence times, we had previously observed that under the model, patrilateral first-cousin mating does not affect X-chromosomal coalescence times, and matrilateral first-cousin mating reduces X-chromosomal coalescence times relative to the nonconsanguineous case; consanguinity produces a greater relative decrease in coalescence times for X chromosomes than for autosomes (Cotter *et al.* 2021, 2022). Owing to the inverse relationship between genomic sharing around a site and the coalescence time at that site (Palamara *et al.* 2012; Carmi *et al.* 2014; Browning SR and Browning BL 2015), corresponding results are reflected in ROH and IBD-sharing calculations under the model. The model predicts longer ROH and IBD-sharing on the X chromosome than on autosomes, owing to three factors: the smaller population size for X chromosomes produces a smaller coalescence time, the stronger effect of matrilateral consanguinity reduces coalescence times to a greater extent relative to the nonconsanguineous model, and reduced recombination in X chromosomes increases ROH and IBD tract lengths.

In accord with this prediction, in data from Jewish populations, we observed that ROH and IBD-sharing did indeed cover a larger fraction of the X chromosome than the autosomes (Fig. 3). Comparing X-chromosomal to autosomal ROH lengths, we observed an increased genomic fraction of ROH on the X-chromosome relative to the autosomes: a 1% increase in autosomal ROH gives rise to a 2.1% increase on the X chromosome (Fig. 4A). For IBD-sharing, a 1% increase in autosomal IBD-sharing predicts a 1.6% increase on the X chromosome (Fig. 4B).

The 2.1% and 1.6% increases on the X-chromosome generally align with model predictions. In a constant-sized population with no consanguinity, our model-based computations found that the ratio of the expected total fractions of the X chromosome and autosomes that lie in ROH or IBD segments approaches 2 for large *N* (equation (5)). In other words, for each 1% increase in the fraction of the autosomal genome covered by ROH or IBD segments, an increase of 2% is predicted for the corresponding coverage of the X chromosome.

We hypothesized that a portion of the increase in X-chromosomal ROH coverage for each 1% increase in autosomal ROH coverage (Fig. 4A) differing from 2% and the corresponding difference from 2% for IBD was attributable to the effects of consanguinity—with matrilateral consanguinity increasing the prediction above 2% and patrilateral consanguinity decreasing it below 2%. This potential attribution is compatible with the observation that the populations studied possess nonzero consanguinity rates, both matrilateral and patrilateral (Table 2). Using equations (8)–(11) to assess the effect of demographic consanguinity rates on ROH X:A ratios directly (Table 3), we see that agreement with predicted ranges is generally closer for ROH than for IBD.

That the empirical analysis generally follows model predictions, with greater sharing on the X chromosome than the autosomes in an amount close to the numerical prediction, supports the value of the model. However, many factors might contribute to deviations of the empirical X-chromosomal and autosomal ROH and IBD patterns from the predictions. First, processes not considered in the model influence differences in genetic variation between X chromosomes and autosomes. For example, differences in the numbers of mating males and females or differing male and female variance of reproductive success can alter effective population size for X chromosomes relative to autosomes (Webster and Wilson Sayres 2016; Cai *et al.* 2023). Further, X–autosome genetic differences can be influenced by various forms of population structure (Wilkins and Marlowe 2006; Ramachandran *et al.* 2008). Recombination differences between X chromosomes and autosomes beyond the $\frac{2}{3}$ we have considered, with different autosomes having different rates per Mb (Kong *et al.* 2002), can affect conversions of $T_{MRCA}$ values to ROH and IBD lengths. These differences can also introduce differences in phasing and ROH and IBD detection; the detection problem is possibly also affected by our use of autosomal ROH cutoffs rather than X-chromosome-specific values in assigning X-chromosomal ROH. In particular, ROH levels might be inflated by use of the autosomal LOD score cutoff for the higher-homozygosity X chromosome.

Beyond these concerns about ROH and IBD detection, a number of limitations may affect our empirical results. Our theoretical analysis relies on centimorgan measurements, whereas we analyze the data in megabases; a more precise comparison of X-chromosomal and autosomal ROH and IBD could be performed by use of a genetic map. The comparison of theoretical and empirical ratios in Table 3 makes use of minimal genomic-sharing cutoffs; we used a cutoff standardized across all theory-based calculations, rather than adding complexity by choosing separate cutoffs for each component of the analysis (ROH vs. IBD, X-chromosomal vs. autosomal, and different populations).

We also note that consanguinity rates are unlikely to be stable over time in real populations, as the model assumes. For example, consanguinity rates from Goldschmidt *et al.* (1960), measured around the mean birth year of the sampled individuals (Kang *et al.* 2016), represent births only at the single time point of 1955–1957; the number of generations over which they would have applied is unclear. Indeed, consanguinity rates have recently declined in some of the sampled populations (Tsafrir and Halbrecht 1972; Cohen *et al.* 2004).

Finally, the data set itself is also limited by a small number of females, so that few data points contribute to inferences on X-chromosomal ROH. We have used these data due to availability of demographic consanguinity rates measured for the four first-cousin types. Additional methodological choices could potentially be investigated in larger genomic data sets in consanguineous populations (e.g. Arciero *et al.* 2021), and an ideal data set would include both large sample sizes as well as demographic estimates of consanguinity.

We have examined how coalescent models and ROH and IBD measurements on the X chromosome and the autosomes can provide information about sex-biased phenomena. Genomic effects of numerous sex-biased processes have been investigated extensively in theoretical models and data, particularly in relation to human populations (Wilkins and Marlowe 2006; Ellegren 2009; Arbiza *et al.* 2014; Goldberg and Rosenberg 2015; Webster and Wilson Sayres 2016). Many organisms possess mating schemes that could induce different kinship levels for autosomes and sex chromosomes (e.g. sex-specific processes and the ZW system in birds, Pizzari *et al.* 2004; Schield *et al.* 2021). As genomic data on ROH and IBD data proliferate in diverse organisms (e.g. Florida scrub jays in Chen *et al.* 2016, dogs in Mooney *et al.* 2021), our approach of examining coalescence times, ROH, and IBD-sharing can potentially contribute to understanding genomic effects of a variety of mating systems.

## Supplementary Material

jkad264_Supplementary_Data

## Data Availability

For the autosomal data, see Kang *et al.* (2016); for the X-chromosomal data, see Behar *et al.* (2013). The demographic data on consanguinity are reported in Goldschmidt *et al.* (1960). Supplemental material is available at G3 online.
