# Supplementary material for: Modeling the effects of consanguinity on autosomal and X-chromosomal runs of homozygosity and identity-by-descent sharing
Source: G3 (Bethesda). 2023 Nov 16;14(2):jkad264. doi: 10.1093/g3journal/jkad264 (PMC10849319; doi:10.1093/g3journal/jkad264)
Supplement: jkad264_Supplementary_Data [file jkad264_supplementary_data.zip › Figure_S2_G3-2023-404651.pdf]

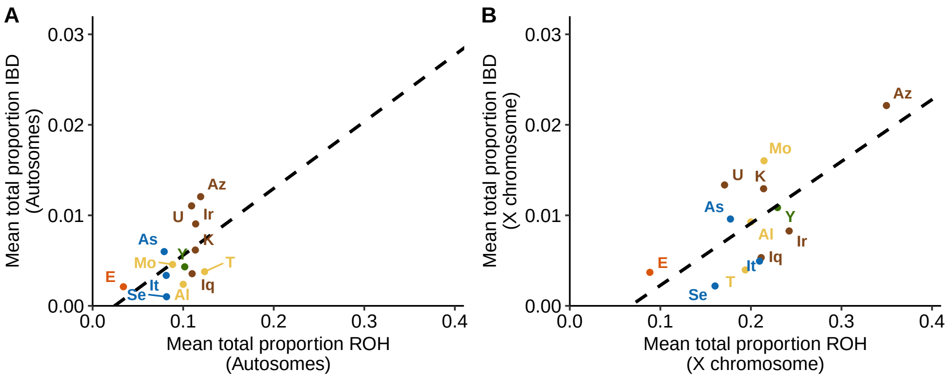

Figure S2: Mean genomic proportion contained in IBD segments versus mean genomic proportion contained in ROH segments. **(A)** Autosomes. **(B)** X chromosome. Thirteen populations are color-coded by regional group as in Kang *et al.* (2016) and Severson *et al.* (2019): Ethiopian, orange; European, blue; Middle Eastern, brown; North African, yellow; Yemenite, green. Population labels: Al, Algerian; As, Ashkenazi; Az, Azerbaijani; E, Ethiopian; Iq, Iraqi; Ir, Iranian; It, Italian; K, Kurdish; Mo, Moroccan; Se, Sephardi; T, Tunisian; U, Uzbekistani; Y, Yemenite. The regression equation is $y = 0.074x - 0.002$ ($R^2 = 0.27$, $P = 0.07$) for autosomes and $y = 0.068x - 0.005$ ($R^2 = 0.49$, $P = 0.008$) for the X chromosome. Both plots use the 13 Jewish populations with ROH data available for the X chromosome.
