# Supplementary material for: Modeling the effects of consanguinity on autosomal and X-chromosomal runs of homozygosity and identity-by-descent sharing
Source: G3 (Bethesda). 2023 Nov 16;14(2):jkad264. doi: 10.1093/g3journal/jkad264 (PMC10849319; doi:10.1093/g3journal/jkad264)
Supplement: jkad264_Supplementary_Data [file jkad264_supplementary_data.zip › Table_S1_G3-2023-404651.pdf]

Population	Females		Males		X data	Demographic data	X *and* demographic data
	**Autosomes**	**X**	**Autosomes**	**X**			
Ashkenazi	5	5	24	22	✓	✓	✓
Iranian	10	9	2	1	✓	✓	✓
Iraqi	5	5	8	5	✓	✓	✓
Libyan	0	0	6	6		✓	
Moroccan	12	10	6	5	✓	✓	✓
Sephardi	5	5	17	14	✓	✓	✓
Syrian	0	0	2	2		✓	
Tunisian	5	5	1	1	✓	✓	✓
Yemenite	12	11	6	4	✓	✓	✓
Algerian	1	1	4	4	✓		
Azerbaijani	4	1	7	7	✓		
Cochin	2	0	5	4			
Ethiopian	14	12	1	0	✓		
Georgian	2	0	5	4			
Italian	3	3	7	7	✓		
Kurdish	3	3	7	6	✓		
Mumbai	0	0	6	4			
Uzbekistani	2	1	3	1	✓		
Total	85	71	117	97			

Table S1: Numbers of sampled individuals in Jewish populations. Values for autosomes correspond to the 202 samples used by Kang *et al.* (2016), and values for the X chromosome correspond to the 168 individuals examined here. The "Demographic Data" column indicates the presence of rate data for the four types of first-cousin consanguinity in Goldschmidt *et al.* (1960)). As in Kang *et al.* (2016), the population listed as "Sephardi" in the table corresponds to the "Turkey" population in Goldschmidt *et al.* (1960), and the population listed as "Iranian" in the table corresponds to the "Persia" population in Goldschmidt *et al.* (1960). Note that quality control procedures differ on the X relative to the autosomes, so that fewer individuals are often available for the X chromosome.
