# Supplementary material for: Modeling the effects of consanguinity on autosomal and X-chromosomal runs of homozygosity and identity-by-descent sharing
Source: G3 (Bethesda). 2023 Nov 16;14(2):jkad264. doi: 10.1093/g3journal/jkad264 (PMC10849319; doi:10.1093/g3journal/jkad264)
Supplement: jkad264_Supplementary_Data [file jkad264_supplementary_data.zip › Table_S2_G3-2023-404651.pdf]

Population	LOD score cutoff
Algerian	0.2687779
Ashkenazi	2.193981
Azerbaijani	0.9832058
Ethiopian	3.118125
Iranian	0.9270845
Iraqi	1.302212
Italian	1.491206
Kurdish	1.101134
Moroccan	1.611013
Sephardi	2.135594
Tunisian	0.6681123
Uzbekistani	-0.0000279
Yemenite	1.710681

Table S2: Log-likelihood (LOD) score cutoffs from Kang *et al.* (2016) used for calling ROH in the 13 Jewish populations with female data.
