# Supplementary figures and images for: Modeling the effects of consanguinity on autosomal and X-chromosomal runs of homozygosity and identity-by-descent sharing
Source: G3 (Bethesda). 2023 Nov 16;14(2):jkad264. doi: 10.1093/g3journal/jkad264 (PMC10849319; doi:10.1093/g3journal/jkad264)

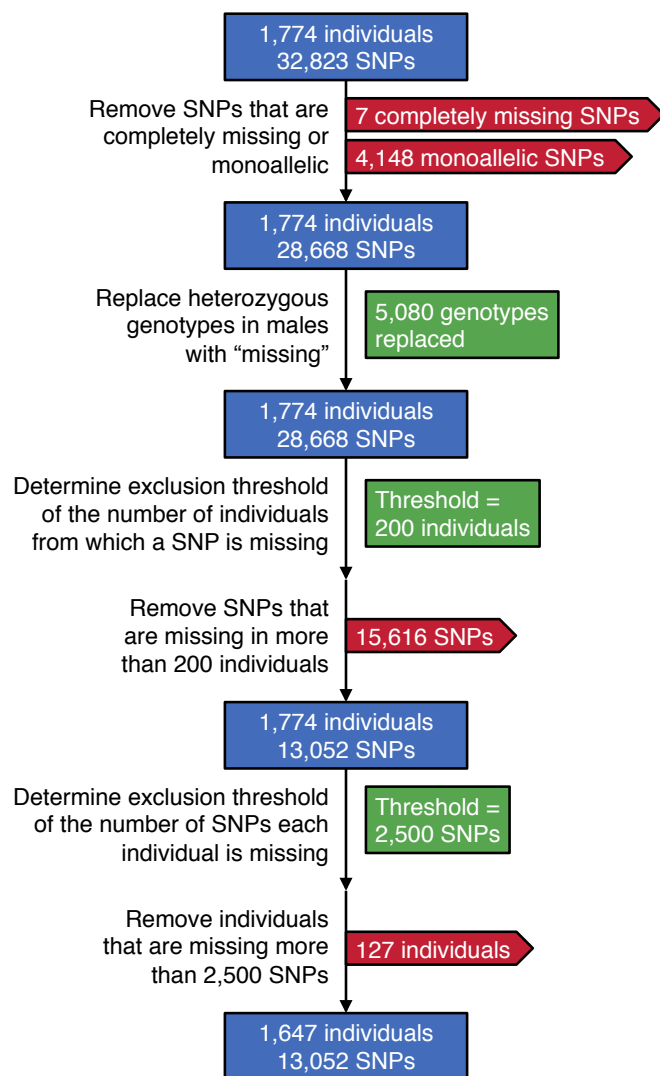

**Figure S1:** Pipeline for processing X-chromosomal data from Behar *et al.* (2013).

Supplement: jkad264_Supplementary_Data [file jkad264_supplementary_data.zip › Figure_S1_G3-2023-404651.pdf]
